# Bioconjugation of a Collagen-Mimicking Peptide Onto Poly(vinyl alcohol) Encourages Endothelialization While Minimizing Thrombosis

**DOI:** 10.3389/fbioe.2020.621768

**Published:** 2020-12-18

**Authors:** Novella M. Bates, Heather E. Heidenreich, Meghan E. Fallon, Yuan Yao, Evelyn K. F. Yim, Monica T. Hinds, Deirdre E. J. Anderson

**Affiliations:** ^1^Department of Biomedical Engineering, Oregon Health & Science University, Portland, OR, United States; ^2^Department of Chemical Engineering, University of Waterloo, Waterloo, ON, Canada

**Keywords:** poly(vinyl alcohol), GFPGER, carbonyldiimidazole, hemocompatibility, vascular graft, platelets, fibrin, endothelial cells

## Abstract

Poly(vinyl alcohol) hydrogel, PVA, is a suitable material for small-diameter vascular grafting. However, the bioinert properties of the material do not allow for *in situ* endothelialization, which is needed to combat common graft failure mechanisms, such as intimal hyperplasia and thrombosis. In this work, the surface of planar and tubular PVA was covalently modified with a collagen-mimicking peptide, GFPGER. The surface of modified PVA was characterized by measuring contact angle and x-ray photoelectron spectroscopy. Endothelial cell attachment to GFPGER-modified PVA was quantified and qualitatively examined using immunohistochemical staining. Then, *in vitro* hemocompatibility testing was performed by quantifying platelet attachment, coagulation factor XII activation, and initiation of fibrin formation. Finally, an established *ex vivo*, non-human primate model was employed to examine platelet attachment and fibrin formation under non-anticoagulated, whole blood flow conditions. GFPGER-modified PVA supported increased EC attachment. *In vitro* initiation of fibrin formation on the modified material was significantly delayed. *Ex vivo* thrombosis assessment showed a reduction in platelet attachment and fibrin formation on GFPGER-modified PVA. Overall, GFPGER-modified PVA encouraged cell attachment while maintaining the material’s hemocompatibility. This work is a significant step toward the development and characterization of a modified-hydrogel surface to improve endothelialization while reducing platelet attachment.

## Graphical Abstract

GRAPHICAL ABSTRACT

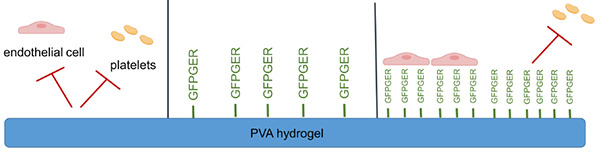

## Introduction

Development of novel, synthetic, small-diameter vascular grafts for treatment of life-threatening cardiovascular diseases has been limited by the challenge of mitigating common failure mechanisms, such as hyperplasia and thrombosis. Currently available and widely used synthetic materials include Dacron^®^ and expanded polytetrafluoroethylene (ePTFE), which are primarily used in bypass surgeries for large diameter (>6 mm) vessels (Nakayama et al., 2004; Kumar et al., 2011; Fayol et al., 2013). Current synthetic materials fail at small diameters, likely due to a mechanical mismatch with native tissues and a lack of an endothelium to promote a healthy, blood-contacting surface. Alternatively, autologous tissues can be used for small-diameter prostheses; however, patients in need of treatment are often critically ill and may not have suitable vessels for this application. Thus, there remains an unmet clinical need for synthetic biomaterials which are hemocompatible and capable of supporting an endothelium.

One synthetic cardiovascular biomaterial under development for vascular tissue engineering applications is poly(vinyl alcohol), PVA, a hydrogel that is amenable to surface modification and has tunable mechanical properties. Previous work thoroughly characterized the mechanical properties of the PVA grafts and found that PVA hydrogels have an elastic modulus ranging from 250 to 500 kPa (Cutiongco et al., 2016a,b), which is similar to the rabbit femoral artery that has a modulus of 230 kPa (Uchida et al., 1989). These hydrogels have a modifiable circumferential compliance, which can mimic native vasculature. PVA compliance ranged between 3 and 7% (Cutiongco et al., 2016a,b), which is comparable to the rabbit femoral artery of 6% (Uchida et al., 1989). Despite PVA having suitable mechanical properties for cardiovascular applications, the bioinert surface, critical for preventing thrombosis, also prevents the material from being endothelialized *in situ* after implantation (Ino et al., 2013). The native vascular endothelium regulates thrombosis and prevents intimal hyperplasia by secreting inhibitory factors, such as nitric oxide and prostacyclin (Liu et al., 2014). Therefore, a widely accepted approach for attaining long term success of vascular grafts is to improve hemocompatibility of synthetic graft surfaces by encouraging *in situ* endothelialization. Chemical modification of bioinert devices like PVA hydrogels, can encourage endothelialization.

To generate a biomimetic tissue engineered surface, the addition of extracellular matrix (ECM)-derived peptides has been shown to increase endothelial cell (EC) attachment. However, a major drawback is that many of these peptides also support unwanted platelet attachment. For decades, work in this area focused on immobilizing peptides based on the Arg–Gly–Asp (RGD) motif in fibronectin; however, there have been mixed reports on platelet adhesion to these sequences (Schmedlen et al., 2002; Li et al., 2008; Sivaraman and Latour, 2010; Gabriel et al., 2011; Cutiongco et al., 2015a; Anderson et al., 2019). Similarly, the hexapeptide, Gly-Phe-Pro-Gly-Glu-Arg (GFPGER), based on the GFOGER motif within collagen, where the O represents hydroxyproline (Xu et al., 2000; Seo et al., 2010), has been studied for its ability to support EC attachment. Both of these sequences specifically recognize α1β1 and α2β1 integrins, which are known to bind ECs and both bind and upregulate platelet activation. The incorporation of GFPGER into materials has been shown to promote cell growth and, while in solution, the GFPGER sequence bound platelets but did not cause activation of the platelets (Munoz-Pinto et al., 2015). The effect on platelet attachment and aggregation due to a covalently linked GFPGER surface is unknown.

Previous work from our group examined the biomimetic effects of mixing ECM proteins and peptides, including GFPGER, into a hydrogel network to enhance EC attachment (Anderson et al., 2019). We found that the GFPGER peptide minimized platelet attachment *in vitro* when compared to PVA-collagen hydrogels. However, antiplatelet monotherapy was required to minimize platelet adhesion under flow in our established *ex vivo*, non-human primate, whole blood, shunt model. This was likely due to the random presentation of amino acids on the hydrogel surface. The possibility of the binding domain being buried within the hydrogel network also suggested the need for a more specific and well-controlled surface binding approach. We hypothesized that covalent attachment of GFPGER to the surface of PVA hydrogels would encourage endothelialization of the material, while mitigating thrombotic responses. The current work presents a systematic examination of this biomimetic tissue-engineered surface for cell attachment and thrombotic potential of PVA hydrogels with GFPGER at various concentrations conjugated to the surface using a 1,1′-carbonyldiimidazole (CDI) linker. This work is a significant step toward the development of hemocompatible off-the-shelf devices for the treatment of cardiovascular disease.

## Materials and Methods

### PVA Film and Tube Manufacturing

Planar PVA films and tubes with an inner diameter (ID) of 4 mm were manufactured, as previously described (Cutiongco et al., 2015b, 2016b). In brief, PVA films were manufactured by adding 15% sodium trimetaphosphate (STMP, Sigma-Aldrich, St. Louis, MO, United States) to aqueous 10% PVA (Sigma-Aldrich) followed by 30% sodium hydroxide. The solution was cast into tissue culture-treated well plates at a 1.5:1 volume to surface area ratio (μL/mm^2^). Films were left to crosslink in a sterile incubator at room temperature with 95% humidity followed by drying in a biosafety cabinet. Films were rehydrated under sterile conditions in 10× phosphate buffered saline (PBS, Fisher Scientific, Waltham, MA, United States) followed by 1× PBS and subsequent deionized water. Prior to modification, samples were dried in an oven overnight. PVA films were then covalently modified with GFPGER (Sigma-Aldrich) peptides. The surface of the PVA films was activated using 100 mg/mL CDI (Sigma-Aldrich) in dimethyl sulfoxide (DMSO, Sigma-Aldrich) for 1 h then briefly rinsed three times with 1× PBS before adding GFPGER (0, 15, 30, 60, and 120 μg/mL) in PBS solutions at 37°C overnight (GFPGER_0_, GFPGER_15_, GFPGER_30_, GFPGER_60_, and GFPGER_120_, respectively). Modified films were rinsed with 1× PBS three times before use.

For the fabrication of tubular PVA samples, a cylindrical mold with an outer diameter of 3.75 mm was coated with a thin PDMS film (Dow Corning, Midland, MI, United States). The mold was treated with air plasma and then was immediately immersed in a solution of crosslinking PVA. Dip casting was performed repeatedly for 12 dips, with a drying duration of 15–30 min between each dip. The tubes were then cured at 18–20°C for 3 days before rehydration in PBS and DI water, as described above, then thoroughly rinsed in DI water overnight. For GFPGER modification, rehydrated and dried PVA tubes were immersed in a 100 mg/mL CDI solution followed by 0, 15, or 120 μg/mL GFPGER solution, as described for the films. Samples were briefly rinsed three times with PBS in between immersions. PVA films were used for surface characterization, cell attachment, and *in vitro* platelet attachment and fibrin formation assays. Tubular samples were used for *ex vivo* thrombosis testing in whole blood.

### Surface Characterization

#### Quantification of Conjugated GFPGER Peptide on the Surface of PVA

Poly(vinyl alcohol) films in a 96-well plate were modified with GFPGER as described above (GFPGER_0_, GFPGER_15_, GFPGER_30_, GFPGER_60_, and GFPGER_120_) with the inclusion of a five percent supplementation of each peptide concentration with fluorescent labeled GFPGER [GFPGER{Lys(5-FAM), GenScript United States, Piscataway, NJ, United States]. The modified films were rinsed with 1× PBS three times before being placed in an Infinite M200 spectrophotometer (Tecan, Männedorf, Switzerland). Fluorescent peptides conjugated to the surface of the PVA films were excited at 488 nm and emission was read at 525 nm. A standard curve of 5% fluorescent-labeled GFPGER at a range of peptide concentrations was used to quantify the peptide on the surface.

#### Captive Bubble Contact Angle

Static contact angle was measured by using a captive bubble method with an optical contact angle system (OCA20, Future Digital Scientific Corp). GFPGER-modified samples were immersed in DI water, and an air bubble (4 μL) was injected into the water with a syringe. The bubble was allowed to attach to the surface of the samples and imaged by a camera. The contact angle on each surface was then calculated using SCA20 software (DataPhysics Instruments United States Corp., Charlotte, NC, United States).

#### X-Ray Photoelectron Spectroscopy

X-ray photoelectron spectroscopy (XPS) was used to obtain the elemental composition on the surfaces of the PVA samples. XPS survey spectra were measured using a VG ESCALab 250 with a monochromatic Al K-alpha X-ray source (1486.6 eV), and high resolution spectra of C1s were obtained using a standard magnesium X-ray source (1253.6 eV) at Waterloo Advanced Technology Laboratory (University of Waterloo, Waterloo, ON, United States).

### Endothelialization of Surfaces

#### Cell Isolation

Carotid arteries were harvested from baboons, and primary endothelial cells (ECs) were isolated, as previously described (Anderson and Hinds, 2012). Artery lumens were filled and incubated for 5 min with 600 U/mL collagenase type II (Worthington Biochemical Corp., Lakewood, NJ, United States) then massaged and flushed into collagen type I (Corning Inc., Corning, NY, United States)-coated tissue culture treated well plates containing VascuLife basal medium (Lifeline Cell Technology, Frederick, MD, United States) supplemented with 18% fetal bovine serum (FBS, Hyclone, Logan, UT, United States). Cell cultures were grown to confluence before sorting with Dynabeads (Invitrogen, Carlsbad, CA, United States) for CD31+ cells per the manufacturer’s protocol. Sorted cells were expanded then stored in freezing media (50% Vasculife basal medium, 40% FBS, and 10% DMSO). Cells thawed for experimentation were maintained in 10% FBS Vasculife basal medium and used at the fourth or fifth passage.

#### Cell Seeding and Quantification

Rehydrated peptide-modified PVA films were incubated in FBS for 1 h at 37°C before seeding ECs at a density of 0.2 M cells/mL in 10% FBS Vasculife basal medium. For cell quantification, cells were cultured for 48 h, rinsed, and frozen dry at −20°C before further lysing with 0.02% sodium dodecyl sulfate in sodium citrate buffer. DNA in the lysate was quantified using a PicoGreen assay per the manufacturer’s protocol (Invitrogen, Carlsbad, CA, United States). Cells were similarly seeded onto tissue culture treated plastic wells to serve as a positive control to the PVA films. Treatment group cell quantities were calculated as a percent confluence from this control.

#### Immunostaining

Cells were incubated on PVA samples for 48 h then stained, as previously described (Jurney et al., 2018). In brief, cells adhered to samples were fixed using 3.7% paraformaldehyde (PFA) then stained for VE-cadherin [primary antibody, Santa Cruz Biotechnology, mouse IgG1 monoclonal, 1:100, 1 h and IgG1 Alexa-488 as a secondary (Invitrogen, goat polyclonal, 1:500, 30 min)], and DAPI (Invitrogen, 1:10000, 5 min) as a nuclear stain. Samples were imaged with a Zeiss LSM 880 inverted confocal microscope system. Low-magnification immunofluorescent (IF) images were collected using a 10× PlanApo objective NA = 0.45. *Z*-stacks are presented in figures as maximum intensity *Z*-projections and post-processing of all images was performed using FIJI (SciJava) (Schindelin et al., 2012; Hagen and Hinds, 2019).

### Thrombogenicity of Surfaces

#### Washed Platelet and Platelet-Rich Plasma Preparation

Human venous whole blood was drawn from healthy donors into sodium citrate, as previously described (McCarty et al., 2005). In brief, whole blood was centrifuged at 200 *g* for 20 min to obtain platelet-rich plasma (PRP). In select studies, purified platelets were isolated from PRP by further centrifugation at 1,000 *g* for 10 min in the presence of 0.1 μg/mL prostacyclin. The pellet was resuspended in HEPES/Tyrode’s buffer (129 mM NaCl, 0.34 mM Na_2_HPO_4_, 2.9 mM KCl, 12 mM NaHCO_3_, 20 mM HEPES, 5 mM glucose, 1 mM MgCl_2_; pH 7.3) containing 0.1 μg/mL prostacyclin. The platelets were washed once via centrifugation (1,000 *g* for 10 min) and resuspended in HEPES-Tyrode’s buffer.

#### Static Platelet Adhesion Quantification

Static platelet adhesion was quantified using a method modified from Vaníčková et al. (2006) to incorporate PVA films. Purified washed platelets suspensions (5.0 × 10^8^ platelets/mL) were incubated on peptide-modified PVA films, in the absence of ECs, in a microtiter plate for 1 h at room temperature. Samples were then rinsed three times with PBS to remove all non-adherent platelets. The amount of platelets attached to the surface of each material was quantified by measuring platelet acid phosphatase activity using a calibration curve of platelet solutions (0–5.0 × 10^8^ platelets/mL).

#### Coagulation Quantification

Pooled platelet-poor plasma (PPP, ISTH SSC Lot 4) was incubated with peptide-modified PVA, in the absence of ECs, films followed by the addition of CaCl_2_ (8 mM). Absorbance at 405 nm was documented over time. Fibrin generation lag times were defined by a 5% increase over baseline absorbance. Rates of fibrin generation were defined by maximum slopes.

Activation of coagulation factor XII (FXII, 200 nM, Haematologic Technologies, Inc., Essex Junction, VT, United States) in the presence of coagulation enzymes generated on GFPGER-modified samples, in the absence of ECs, was measured in a purified system using a chromogenic substrate, Spectrozyme-FXIIa (American Diagnostica, Inc., Stamford, CT, United States), as previously described (Bates et al., 2020).

#### Whole Blood Platelet and Fibrin Adhesion Under Flow Quantification

Rehydrated GFPGER-modified tubes, in the absence of ECs, (prepared from 15 or 120 μg/mL GFPGER solutions) were incorporated into a chronic, femoral arteriovenous (AV) shunt via connective silicone tubing in an established non-anticoagulated, non-human primate model (Cutiongco et al., 2015a; Anderson et al., 2019). Juvenile male baboons (9–12 kg) underwent a minor surgical procedure, cannulation of the superficial femoral artery and vein, for the placement of a chronic AV shunt. The AV shunts were composed of implanted sterilized silicone rubber tubing (3.0 mm ID, SIL-TEC, Technical Products, Inc, Lawrenceville, GA, United States). Single devices were connected to the implant with Silastic tubing (4 mm ID, Dow Chemical, Midland, MI, United States) (Supplementary Figure 1). Upstream of the device was a flow probe (Transonic, Ithaca, NY, United States) to measure blood velocity, and downstream of the device was a tubing clamp to control the flow at a steady rate. Autologous platelets and homologous fibrin were labeled with indium-111 (^111^In) and iodine-125 (^125^I), respectively. Samples were subjected to whole blood flow at 100 mL/min and platelet accumulation onto the samples was quantified in real-time by measurement of ^111^In radiation using a Brivo NM615 nuclear imaging camera (General Electric, Boston, MA, United States). The amount of fibrin was quantified as an end-point measurement of ^125^I radiation approximately 30 days post experiment, once the ^111^In decayed, using a 1480 Wizard gamma counter (PerkinElmer, Waltham, MA, United States). CDI-active PVA without peptide (GFPGER_0_) and plain PVA were used as negative controls, collagen-coated ePTFE (Collagen, 1 mg/mL equine collagen type I, Chrono-log Corp.) was used as a positive control and clinical grade ePTFE (IMPRA^®^, Bard Peripheral Vascular, Inc., Tempe, AZ, United States) with an ID of 4mm was used as a clinical control.

After whole blood testing, the tubes were rinsed with PBS and fixed with 3.7% PFA for 48–72 h before additional rinsing and storage. Some of the samples used in the shunt studies were examined with micro-computed tomography (MicroCT). The samples were prepared as described previously (Gupta et al., 2020). Briefly, PVA samples were filled with Microfil^®^ polymer to render the lumens radiopaque. Since the Microfil^®^ polymer is incompatible with ePTFE, ePTFE samples were soaked in Lugol’s (Sigma-Aldrich) solution to render the thrombus radiopaque.

Shunt studies were performed in a male *Papio anubus* baboon (10.5 kg), which was under the care of the Oregon National Primate Research Center (ONPRC) staff. All studies abided by guidelines provided by the National Research Council and the Committee on Care and Use of Laboratory Animals of the Institute of Laboratory Animal Resources. All studies were approved by the ONPRC Institutional Animal Care and Use Committee.

#### Micro-Computed Tomography Analysis

Micro-computed tomography (Inveon, Siemens) imaging was used to analyze thrombus area over the length of the samples (volume) in the small-diameter vascular grafts formed under flow by whole blood in *ex vivo* shunts, as described previously (Gupta et al., 2020). Imaging was performed with 2 × 2 binning, 220 projections, at an exposure of 660 ms/projection. Thrombus physical characteristics were determined with the Amira^®^ software package (FEI, version 5.2.2) by a trained operator blinded to specific sample treatments. The software also generated three-dimensional (3D) volume renderings of either the thrombus for ePTFE grafts or the lumen for collagen-coated and PVA grafts. The software-calculated volumes and lengths of each sample were used for subsequent analysis. Amira^®^ was utilized, as previously described (Gupta et al., 2020), to segment specific regions of interest (ROIs) in each cross-sectional image. For PVA samples, the ROI was the luminal area of the graft. ePTFE samples were segmented for the thrombi as the ROI, and collagen-coated ePTFE samples contained ROIs of both the luminal and thrombus areas. 3D representations of either the lumen (PVA and collagen-coated ePTFE) or thrombus (ePTFE) of the grafts were generated from ROI summations, and the surface areas of each cross-sectional slice were subsequently measured. These cross-sectional data were then utilized in analyzing average thrombus generation over the entire graft surface. The cross-sectional areas obtained from each slice were used to generate topography maps of the thrombus surface for each individual sample. This allows for characterization of the thrombus surface and quantification of the intrasample variability of the thrombus over the length of a single sample.

### Statistical Methods

Data are presented as mean ± standard deviation (SD) for all studies. Probability values of *p* < 0.05 were considered to be statistically significant. To determine statistical significance of captive bubble contact angle (*n* = 3), XPS (*n* = 3), peptide quantification (*n* = 5), lag time and rate of fibrin generation (*n* = 3), static *in vitro* platelet adhesion (*n* = 3), and *ex vivo* end-point fibrin accumulation data (*n* = 6), one-way analysis of variance (ANOVA) tests were performed. To determine statistical significance for *ex vivo* platelet data, a repeated measures ANOVA was performed on surface modified PVA samples (*n* = 6). Platelet reactivity of collagen-ePTFE, ePTFE, and unmodified PVA controls samples (*n* = 2) were confirmed and compared to historic data, but these controls were not included in the statistical analysis. All studies were tested for ANOVA assumptions using Levene’s and qqplots and, when necessary, data were natural log transformed. A subset of the *ex vivo* samples were tested with microCT (*n* = 4 for GFPGER_0_, GFPGER_15_, and GFPGER_120_) using a one-way ANOVA between the average luminal cross-sectional area (volume/length) for each test group. SDs of the volume data generated over the entire graft length for each single sample (generated from per slice area data) were also compared with a one-way ANOVA to determine if intrasample variability was significantly different between test groups.

## Results

### Surface Characterization

#### Peptide Quantification

**FIGURE 1:**
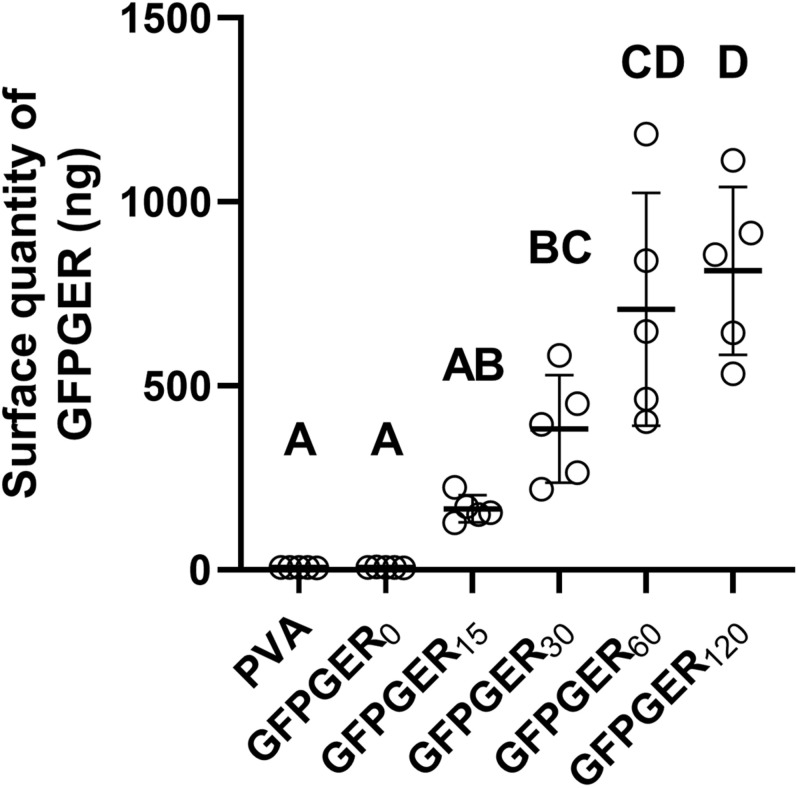
Quantification of GFPGER peptide on the surface of modified PVA samples. The quantity of immobilized GFPGER peptide on the surface of each PVA sample was determined by fluorescent peptide supplementation and is shown as mean ± SD (*n* = 5). Statistical analysis was performed using a one-way ANOVA test with Tukey’s HSD *post hoc* testing. Groups which do not share a letter indicate statistical significance (*p* < 0.05).

Gly-Phe-Pro-Gly-Glu-Arg peptides were supplemented with 5% fluorescent-labeled peptide to quantify the amount of GFPGER immobilized onto the surface of PVA at each concentration. A significant increase in peptide quantity was found on GFPGER_30_, GFPGER_60_, and GFPGER_120_ when compared to unmodified samples (Figure 1). Additionally, GFPGER_60_ and GFPGER_120_ were significantly increased from GFPGER_15_, and GFPGER_120_ was significantly increased from GFPGER_30_.

#### Contact Angle

**FIGURE 2:**
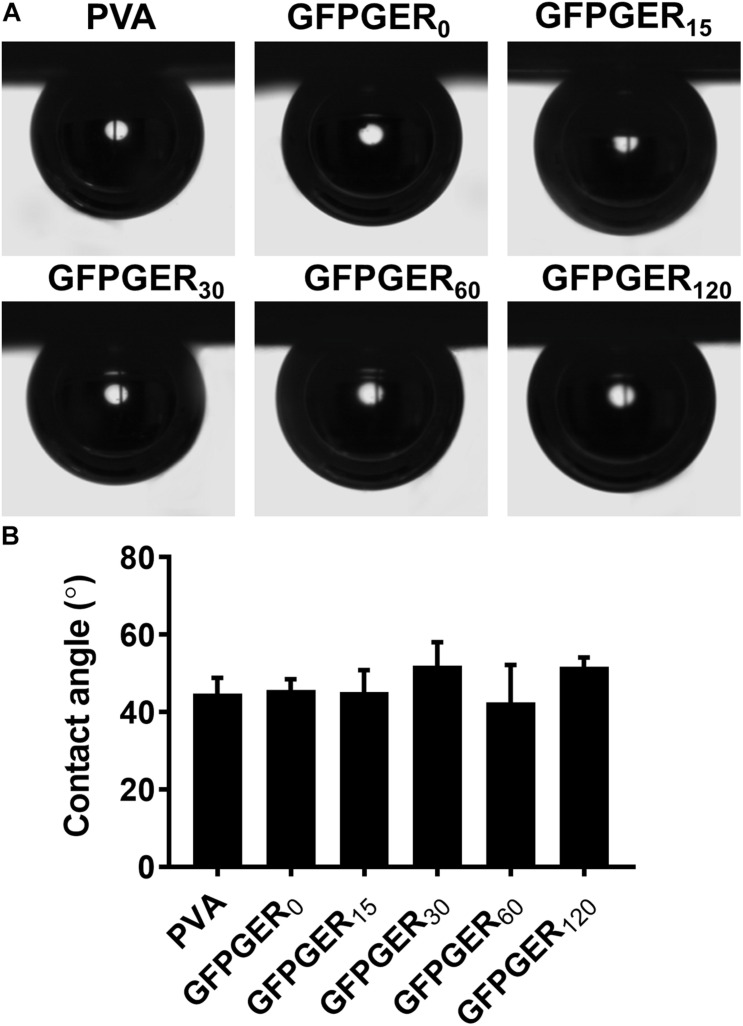
Contact angle of PVA films modified with different concentrations of GFPGER. The contact angle was measured using a captive bubble method. Representative images **(A)** and contact angle data (*n* = 6) are shown as mean ± SD **(B)**. Statistical analysis was performed using a one-way ANOVA test. No significant difference was observed among all groups.

Contact angle did not exhibit a significant difference among all test groups (Figure 2). The surfaces of PVA and GFPGER modified PVA samples all remained hydrophilic, with contact angles ranging from 44.8 to 51.7°.

#### XPS

**FIGURE 3:**
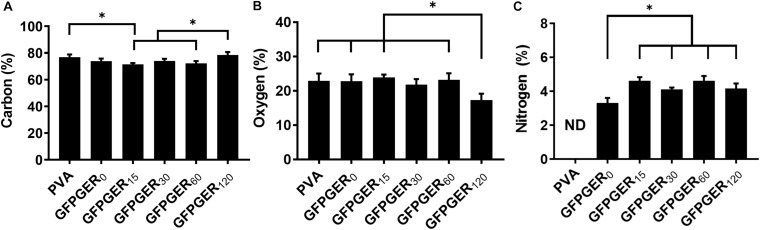
XPS measured atomic percentages of PVA films modified with different concentrations of GFPGER. XPS was used to measure the percentage of carbon, oxygen, and nitrogen on GFPGER-modified surfaces. Atomic percentages (*n* = 3) are shown as mean ± SD for all samples for carbon **(A)**, oxygen **(B)**, and nitrogen **(C)**. Statistical analysis was performed using a one-way ANOVA with Tukey’s HSD *post hoc* testing. ^∗^indicates statistical significance (*p* < 0.05). ND = not detected.

X-ray photoelectron spectroscopy survey spectra of PVA and GFPGER modified PVA are shown in Supplementary Figure 1A. GFPGER and CDI contain nitrogen and, thus, all modified PVA groups showed significant N1s peaks. Table 1 and Figure 3 showed the atomic percentage of C, O, and N on different samples. GFPGER_120_ had significantly higher C% than GFPGER_30_ and GFPGER_0_, and significantly lower O% than all the other groups. GFPGER modified PVA samples had significantly higher N% than GFPGER_0_. Supplementary Figures 2B–D shows the high-resolution spectra of carbon on PVA, GFPGER_0_, and GFPGER_120_ samples. High-resolution spectrum of carbon on PVA showed characteristic peaks of the PVA structure at 282, 284, and 286 eV from C–C/C–H, C–O, and C=O, respectively. In addition to the characteristic PVA peaks, GFPGER_0_ also showed a peak at 288 eV from the –O–C=O bond in the imidazole carbamate intermediate groups. GFPGER contains NH-C(=O) groups, and the peak at 288.7eV observed from GFPGER_120_ samples further confirmed the presence of GFPGER on the samples.

**TABLE 1:** XPS results of PVA films modified with different concentration of GFPGER.

**Atomic %**	**C**	**O**	**N**
PVA	76.8 ± 2.0	23.1 ± 2.0	ND
GFPGER_0_	73.9 ± 1.9	22.7 ± 2.1	3.3 ± 0.3
GFPGER_15_	71.4 ± 1.0	23.9 ± 0.8	4.6 ± 0.2
GFPGER_30_	74.0 ± 1.6	21.8 ± 1.6	4.11 ± 0.1
GFPGER_60_	72.2 ± 1.7	23.2 ± 1.9	4.6 ± 0.3
GFPGER_120_	78.6 ± 2.2	17.3 ± 1.9	4.17 ± 0.3

*Atomic percentage of carbon, nitrogen, and oxygen. Data (*n* = 3) are shown as mean ± SD (ND = not detected).*

### Endothelialization of Surfaces

**FIGURE 4:**
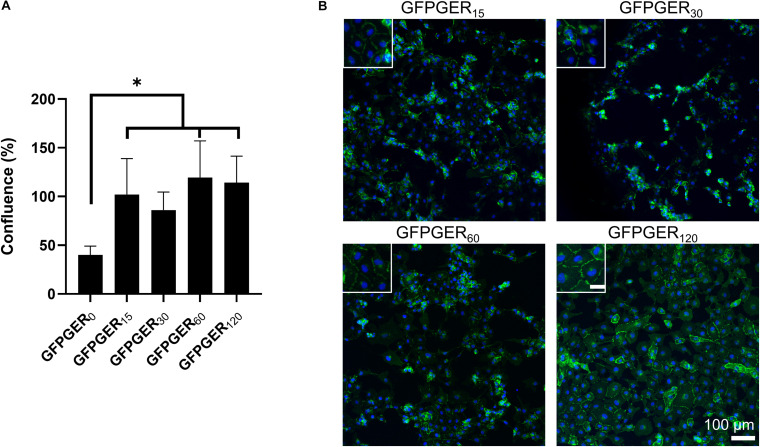
EC quantification and visualization after 48 h on GFPGER-conjugated PVA. Cell attachment increased on modified PVA compared to unmodified PVA **(A)**. Data (*n* = 4–6) are presented as a percent increase from ECs adherent on a tissue culture plastic (TCP) control, where ECs were 100% confluent. Statistical analysis was performed using a one-way ANOVA with Tukey’s HSD *post hoc* testing. ^∗^indicates statistical significance (*p* < 0.05). Representative staining of baboon carotid ECs on GFPGER-conjugated PVA films after 48 h showed adherent ECs on all modified PVA samples, while no cells were observed on unmodified PVA **(B)**. Green = VE-cadherin (primary antibody and IgG1 Alexa-488 as a secondary) and blue = DAPI as a nuclear stain. Inset scale bar = 30 μm.

We examined the ability of PVA to attach ECs after the hydrogel was modified with GFPGER peptide. ECs that adhered to samples were quantified using PicoGreen^®^ as a percentage of cells adhered to tissue culture plastic and observed qualitatively using fluorescent staining. Only the GFPGER-modified samples (15, 60, and 120 μg/mL) showed a significant increase in EC attachment when compared to GFPGER_0_ (Figure 4A). These results were also reflected qualitatively in the obtained stained images (Figure 4B). GFPGER_120_ showed particularly robust VE-cadherin staining suggestive of strong cell-cell interactions. Adherent ECs were not detected nor observed on plain PVA samples, similar to results from our work (Jurney et al., 2018; Anderson et al., 2019).

### Thrombogenicity of Surfaces

#### Static *in vitro* Platelet Adhesion Quantification

**FIGURE 5:**
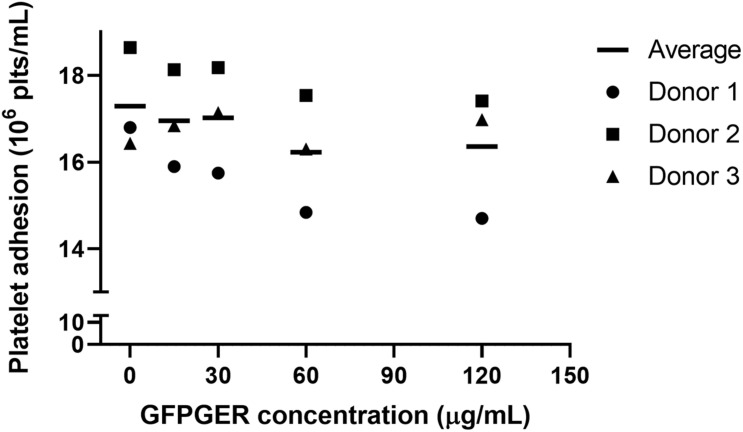
Quantification of static platelet adhesion of purified platelets onto GFPGER-conjugated PVA samples. Platelet adhesion after 1 h, under static, purified conditions, was measured on the surface of GFPGER-modified PVA samples. Data (*n* = 3) are shown as individual biological replicates with means overlaid. Statistical analysis was performed using a one-way ANOVA test. No significant difference was observed among all groups.

We studied the extent to which peptide-modified PVA samples supported static platelet adhesion using a solution of purified human platelets *in vitro*. There were no statistical differences found (Figure 5).

#### Fibrin Clotting Time, Rate of Fibrin Generation *in vitro*, and FXII Activation

**FIGURE 6:**
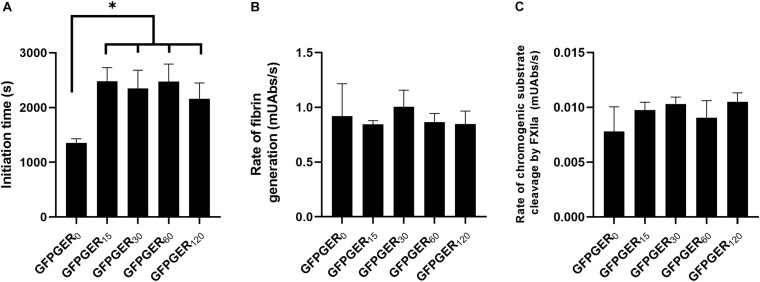
Quantification of *in vitro* coagulation on GFPGER-conjugated PVA samples. Coagulation potential of GFPGER-modified samples was determined by measuring fibrin initiation time, rate of fibrin generation, and activity of coagulation factor XII from static coagulation assays. Fibrin initiation lag times in platelet-poor-plasma in the presence of GFPGER-conjugated PVA samples showed prolonged initiation times on GFPGER-modified samples **(A)**. No difference in rate of fibrin generation on GFPGER-conjugated PVA samples was measured **(B)**. There was no difference in FXIIa produced by GFPGER-conjugated PVA samples, shown as rate of cleavage of a chromogenic substrate, when compared to unmodified **(C)**. Data (*n* = 3–6) are shown as mean ± SD. Statistical analysis was performed using a one-way ANOVA with Tukey’s HSD *post hoc* testing. ^∗^indicates statistical significance (*p* < 0.01).

We quantified the time to fibrin clot formation in PPP and the rate of fibrin formation on peptide-modified PVA samples. GFPGER-modified samples all had prolonged times to initiation of fibrin generation when compared to CDI only-modified samples (Figure 6A). There were no significant differences observed in the rate of fibrin generation in the presence of GFPGER-modified PVA films when compared to the baseline (Figure 6B).

To determine if the contact pathway was playing a role in the difference of time to initiation of fibrin formation, activation of FXII was measured (Figure 6C). There were no significant differences observed between unmodified and GFPGER-modified PVA samples.

#### Whole Blood Platelet and Fibrin Adhesion Under Flow Quantification

**FIGURE 7:**
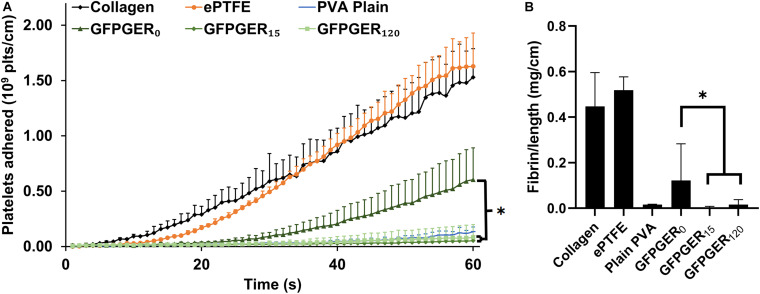
Whole blood, *ex vivo* shunt testing. Tubular GFPGER-modified PVA (prepared from 15 or 120 μg/mL GFPGER solutions, *n* = 6) and unmodified but CDI-active PVA (*n* = 6) were tested in an *ex vivo* shunt model. Collagen-coated ePTFE (*n* = 2), ePTFE (*n* = 2), and plain PVA (*n* = 2) were run to compare to historical control data. **(A)** Platelet accumulation was quantified over 60 min exposure to whole blood. Data are shown as mean ± range or SD. Statistical analysis was performed only on the modified PVA samples using a repeated measures ANOVA with Tukey’s HSD *post hoc* testing. GFPGER_0_ was found to be significant from GFPGER_15_ (*p* < 0.001) and from GFPGER_120_ (*p* = 0.001), while GFPGER_15_ was not significant from GFPGER_120_. **(B)** Fibrin data were quantified and normalized per centimeter of axial length for all grafts at 60 min. Data (*n* = 2–6) are shown as mean ± range/SD. Statistical analysis was performed using a one-way ANOVA with Tukey’s HSD *post hoc* testing (*p* < 0.001). ^∗^indicates statistical significance.

We then examined platelet and fibrin deposition onto GFPGER-modified tubes from whole, flowing blood with *ex vivo* thrombosis testing. We observed a significant decrease in platelet attachment onto GFPGER-modified samples when compared to the CDI only-modified samples for both concentrations of tested conjugated peptide, low (15 μg/mL) and high (120 μg/mL) (Figure 7A). Unmodified PVA samples were also quite low compared to the positive and clinical controls and consistent with both concentrations of GFPGER modifications. We observed a similar significant decrease in fibrin formation on GFPGER_15_ and GFPGER_120_ when compared to the CDI-only samples (Figure 7B).

#### MicroCT

**FIGURE 8:**
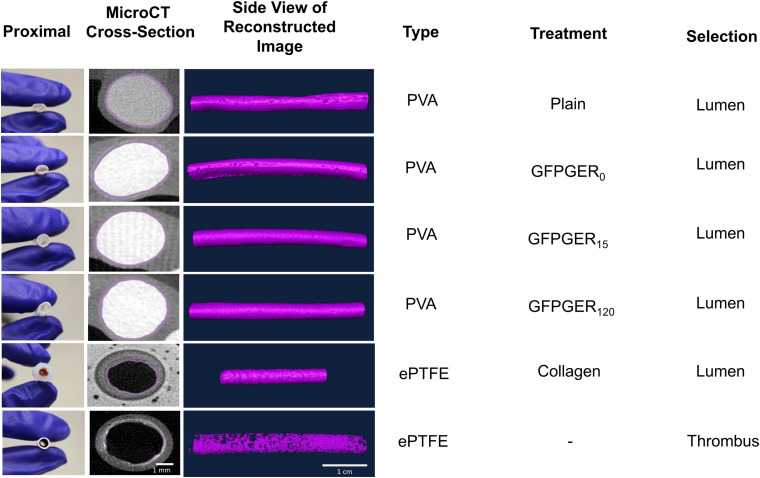
Three-dimensional surface volume renderings of microCT cross-sectional ROI selections using Amira^®^ showing thrombi in the lumen of tested samples. All grafts from *ex vivo* whole blood testing were 4–5 mm ID. PVA and ePTFE samples were 4 cm long and collagen-coated ePTFE samples were 2 cm long. ROI selection from a blinded, trained user is shown in purple, designating the user-selected masking range. Scale bar for microCT cross-sectional images and volume renderings are 1 mm and 1 cm, respectively.

**FIGURE 9:**
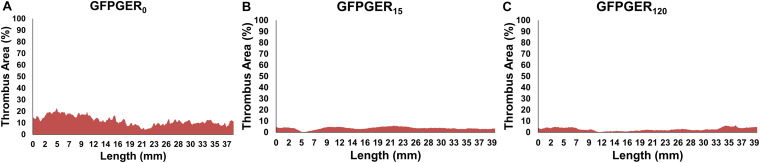
Quantification of the cross-sectional thrombus area as a percentage of the maximal luminal area per image slice over the entire graft length for a representative sample of GFPGER_0_ **(A)**, GFPGER_15_ **(B)**, and GFPGER_120_ **(C)**. Samples illustrate the variability seen in thrombus over the length of a single graft after 1 h of whole, flowing blood. Samples conjugated with GFPGER **(B, C)** had less thrombus and less observed variability than the control sample with only a vehicle control **(A)**.

**FIGURE 10:**
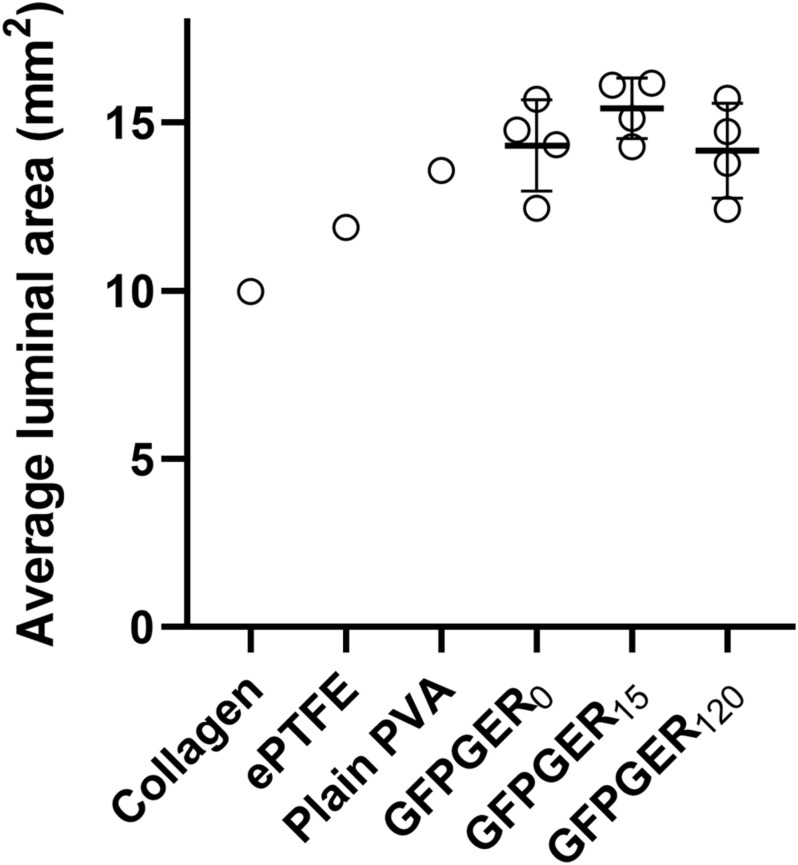
Average luminal area of grafts after exposure to whole flowing blood in the *ex vivo* shunt. Collagen (*n* = 1), ePTFE (*n* = 1), plain PVA (*n* = 1), GFPGER_0_, GFPGER_15_, and GFPGER_120_ (all covalently modified GFPGER PVA samples were *n* = 4) grafts were analyzed for the average cross-sectional area per slice of the lumen. Data are presented as the mean ± SD of the average luminal area. Statistical analysis was performed only on experimental (GFPGER) samples using a one-way ANOVA. No statistical differences were observed.

Three-dimensional renderings of either the lumen (PVA and collagen-coated ePTFE) or thrombus (ePTFE) area along the length of the samples (volume) were generated based on cross-sectional ROI segmentation of each graft type (Figure 8). The measurement of thrombus area along the length of each individual graft as a percentage of the maximal luminal area was evaluated and representative samples are shown in Figure 9. This representative GFPGER_0_ sample (Figure 9A) had an average thrombus area percentage of 12.10 ± 3.99% of the maximal luminal area, the representative GFPGER_15_ sample (Figure 9B) had an overall thrombus area percentage of 3.66 ± 1.11%, and the representative GFPGER_120_ sample (Figure 9C) had an overall thrombus area of 2.68 ± 1.41%. By capturing the area per sample slice, this method of analysis allows for the study of variability of an individual thrombus. The intrasample variability was quantified as the standard deviation of the individual thrombus area as a percent of the maximal luminal area of each sample type. These standard deviations were averaged and compared between all the PVA samples (*n* = 4) resulting in variabilities of GFPGER_0_ (4.17 ± 0.87%), GFPGER_15_ (3.10 ± 2.84%), and GFPGER_120_ (4.26 ± 2.39%). These variability data were not statistically significant. The average Amira^®^ luminal area for each type of PVA modifications (*n* = 4) were also compared and showed no statistical significance between the three GFPGER-modified groups (Figure 10).

## Discussion

Successful integration of synthetic small-diameter vascular grafts *in vivo* is impeded by a lack of *in situ* endothelialization and material-vessel compliance mismatch leading to neointimal hyperplasia and thrombosis and potential loss of patency. Studies of compliant PVA biomaterials suggest that the low thrombogenicity of the material makes it a promising vascular graft material (Chaouat et al., 2008; Cutiongco et al., 2015a,b; Jurney et al., 2018; Anderson et al., 2019; Rizwan et al., 2020; Yao et al., 2020). However, in the absence of surface modifications, PVA is unable to support EC attachment and growth. We hypothesized that the covalent attachment of a collagen-mimicking peptide to PVA would enhance EC adhesion, without compromising hemocompatibility of the material. Thus, we modified PVA hydrogel surfaces using CDI chemistry with the collagen-mimetic peptide, GFPGER, a low-cost, commercially available peptide, which specifically recognizes α1β1 and α2β1 integrins. We performed characterization of this modified surface as well as thorough *in vitro* and *ex vivo* hemocompatibility testing to assess the thrombotic potential of the material.

Using CDI as a crosslinker enabled a concentration dependent increase in GFPGER covalently bound to the PVA surface. Upon examination of the surface chemistries present on the modified hydrogels, we found an increase in C and N%, which indicated the introduction of amine groups onto the hydrogel, while the contact angle remained unchanged when compared to unmodified PVA. CDI has been used as a chemical crosslinker to modify the surface of polymers and hydrogels, including PVA, with full length proteins (Nuttelman et al., 2001; Rizwan et al., 2020; Yao et al., 2020). CDI reacts with hydroxyl groups to form highly reactive, anchoring carbamate groups, which are then available to react with proteins or peptides for immobilization. Nuttelman et al. (2001) have previously used CDI to covalently attach fibronectin, an ECM protein found to play a role in cell adhesion and proliferation, to PVA. In their work, an 11-carbon spacer, bromoundecanoic acid, was added to the PVA hydrogel in an intermediate step before CDI activation, followed by the addition of fibronectin. Using this chemical scheme, they found that fibroblast cell attachment, proliferation, and migration were all significantly increased on fibronectin-conjugated PVA when compared to the unmodified material. However, the additional acid wash step is cumbersome and unnecessary, as shown by Rizwan et al. (2020), in which CDI was used to covalently attach gelatin, another cell-adhesive protein, directly to PVA. In this prior study, CDI alone was used to aminate the surface of PVA before protein modification and the presence of gelatin on the PVA surface after the covalent modification was confirmed using Fourier transform infrared spectroscopy and XPS. Their surface characterization results showed a significant increase in nitrogen on the surface, which, like the results in the current study, indicate the presence of amine groups on the material surface. While some peptides, such as REDV (Lei et al., 2012), require spacers in order to retain their bioactivity, we successfully grafted GFPGER to PVA using CDI and the peptide remained active. Additionally, it is unlikely that using CDI chemistry to immobilize peptides to the surface of PVA would alter the mechanical properties of the material as was shown by previous work from our group (Rizwan et al., 2020), whereas there is more potential for physical alteration of the material when mixing peptides into the hydrogel network. To our knowledge, the work presented here is the first time that CDI has been used to covalently attach an ECM-mimicking peptide to the surface of PVA.

In our cell studies, we found that GFPGER-conjugated PVA supported the formation of an endothelial layer. This result is similar to cell attachment results obtained in our previous GFPGER-mixed PVA studies, where we found greater cell adhesion to GFPGER-mixed PVA samples when compared to PVA modified with collagen and other proteins and peptides (Anderson et al., 2019). In the current study, EC adhesion to most concentrations of GFPGER–modified PVA was significantly increased when compared to the CDI-only PVA baseline (GFPGER_0_), and a confluent layer of ECs was seen at the lowest and highest concentrations of GFPGER-conjugated samples. Despite the surface characterization which suggested that the GFPGER had not saturated the surface at the lower concentrations, it was interesting to see that all the examined concentrations had substantially increased cell adhesion that was not significantly different from other GFPGER concentrations. Plain PVA is biologically inert and does not support cell attachment alone (Cutiongco et al., 2015a, 2016b; Rizwan et al., 2020). The EC attachment attained is likely through the α1β1 and α2β1 integrins, which are well known to interact with GFPGER (Seo et al., 2010). This cell attachment is in agreement with the work of several groups, including ours (Chaouat et al., 2008; Munoz-Pinto et al., 2015; Anderson et al., 2019). GFPGER has been shown to support cell attachment and spreading on several surfaces, including a collagen-mimetic protein with GFPGER incorporated, Scl2_GFPGER_, coated onto tissue culture plastic (Seo et al., 2010) and a Scl2_GFPGER_ incorporated PEG hydrogel (Cereceres et al., 2015). However, we were able to successfully conjugate GFPGER alone directly to a hydrogel surface via a simple chemical scheme, eliminating the need for site-directed mutagenesis or in-house protein synthesis.

Endothelial cells have been shown to have a distinct cobblestone appearance when cultured *in vitro* (Glynn and Hinds, 2014). Despite being cultured under identical conditions, the morphology of ECs on the different densities of GFPGER-conjugated PVA varied. ECs on GFPGER_15_ were less spread and dominated by their nucleus whereas cells on GFPGER_120_ appear to have spread more with a larger cytoplasm and the characteristic VE-Cadherin expression of *in vitro* cultured confluent ECs. The difference in peptide density was also demonstrated by the distinct cell wall borders observed around cells on hydrogels with the highest concentration of GFPGER. The increase in confluency and altered morphology on GFPGER_120_ indicates that higher peptide concentrations may lead to phenotypic alteration of ECs. Hydrogels with a higher density of peptide have more binding sites for EC integrins, which encourages EC spreading to a greater degree than lower densities of EC adhesive peptide. However, this phenomenon was not observed in GFPGER-mixed PVA studies (Anderson et al., 2019); thus, more studies would need to be performed to understand the relationship between conjugated-peptide density and EC morphology and phenotype.

Importantly, the CDI-GFPGER modification of PVA did not increase platelet attachment and aggregation nor did the modification initiate clotting via the coagulation cascade. In our evaluation of GFPGER-conjugated PVA hydrogels, we found that all GFPGER-conjugated samples delayed the initiation time of fibrin clotting of PPP and decreased the fibrin formation *ex vivo* when compared to the baseline material (GFPGER_0_). The lack of a significance difference found in the analysis of FXII activation on PVA samples suggested that contact pathway was not a potential coagulation initiation mechanism. However, more studies will have to be done to determine the mechanism of fibrin formation inhibition on GFPGER-modified samples. *In vitro* and *ex vivo* assessment of platelet attachment onto GFPGER-conjugated samples indicated no alteration of the PVA hemocompatibility. MicroCT analysis of thrombi formed on PVA samples after *ex vivo* testing showed minimal thrombus formation. These results contrast what is known about collagen-platelet interactions (Alberio and Dale, 1998; Farndale, 2006; Ruggeri and Mendolicchio, 2007; Harrison et al., 2011) and our previous GFPGER-mixed PVA studies (Anderson et al., 2019), where we found significantly higher platelet attachment to GFPGER-mixed samples when compared to unmodified PVA. The GFPGER binding site to α2β1 integrin on platelets is known to contribute to platelet activation and aggregation (Cosemans et al., 2008; Munnix et al., 2008). The divalent cation in the α2 I-domain of the integrin coordinates with the glutamate in collagen or collagen-mimicking peptides, leading to a conformational change in the receptor followed by activation. Rich et al. (1999) predicted that the ligand-binding site in the α1 subunit, which is found on ECs, is longer and more flexible than the α2 subunit (Xu et al., 2000), which is present on the platelet surface. Previous studies found that GFPGER has slightly less affinity for the α2 subunit, when compared to GFOGER, the native integrin binding sequence in collagen (Sipilä et al., 2018). This decreased affinity may be enough to minimally bind platelets and not activate key pathways leading to aggregation. Additionally, when GFPGER is directly bonded to the surface of PVA, as with the CDI linker, the glutamate that participates in integrin coordination is always available, leading to a controlled interaction between the material and biological components. Mixing the peptide into the PVA, as was done previously (Anderson et al., 2019), buries some of the key groups needed for integrin recognition, which likely led to non-specific binding of both cells and platelets (Anderson et al., 2019). Thus, CDI-mediated binding of GFPGER is a promising surface modification of blood contacting materials, which does not activate platelets.

Ultimately, the work presented herein supported our hypothesis that covalent attachment of GFPFER to the surface of PVA hydrogels would encourage endothelialization of the material, while attenuating thrombosis. We presented a systemic characterization and evaluation of GFPGER-conjugated PVA hydrogels using *in vitro* and *ex vivo* experimentation. We have shown that covalently modifying PVA with a low cost, widely available commercial peptide, GFPGER, for use as a synthetic vascular graft material may prevent thrombosis, while promoting *in situ* endothelialization of the material. A limitation of this work is the short time duration in which thrombogenesis is studied. Future work will include longer shunt study times and implantation studies using established rabbit (small animal) carotid bypass grafting (Cutiongco et al., 2016b) and non-human primate (large animal) aortoiliac bypass grafting (Anderson et al., 2018) implant models both with an end-to-side methodology to increase pre-clinical understanding of material integration. These models provide the best approach for recapitulating clinical flow dynamics, which is frequently neglected in other vascular graft animal models (Zilla et al., 2007). Additionally, further *in vitro* work will be done to investigate specific EC phenotype and SMC proliferation on GFPGER-conjugated PVA. The promising results reported here with our novel synthetic biomaterial support the potential of this covalently linked surface modification either to coat off-the-shelf cardiovascular materials or for tissue-engineered constructs, supporting endothelialization without aggregating platelets.

## Data Availability Statement

The raw data supporting the conclusions of this article will be made available by the authors, without undue reservation.

## Ethics Statement

The animal study was reviewed and approved by the ONPRC Institutional Animal Care and Use Committee.

## Author Contributions

NB performed *in vitro* thrombogenicity studies, peptide quantification studies, analyzed data, wrote and prepared the manuscript/figures for publication. HH performed *in vitro* thrombogenicity studies and endothelial cell attachment studies. MF performed microCT studies, associated analysis, and contributed to writing and preparing the manuscript/figures. YY performed surface characterization work, prepared tubular samples for arteriovenous shunt studies, analyzed data, and contributed to writing and preparing the manuscript/figures. EY and MH contributed to the original idea, experimental design, scientific guidance, discussions, laboratory space, and funding. DA contributed to the original idea, experimental design, scientific guidance, discussions, funding, *in vitro* thrombogenicity studies, endothelial cell attachment studies, and contributed to writing and preparing the manuscript/figures. All authors revised the manuscript.

## Conflict of Interest

The authors declare that the research was conducted in the absence of any commercial or financial relationships that could be construed as a potential conflict of interest.

## Supplementary Material

Supplementary Figure 1 Shunt study set up. A shunt was placed between the femoral artery and the femoral vein of a non-human primate (not pictured) using Silastic tubing. The sample to be tested was placed between the tubing using connectors, such that blood flow was continuous through the *ex vivo* shunt loop. Whole blood was used in this study without the presence of anticoagulant or antiplatelet therapies. Flow rate was controlled at 100 mL/min using the downstream flow clamp and measured using the upstream flow probe. This figure has been modified to remove all informal labeling using Photoshop.

Supplementary Figure 2 **(A)** Survey XPS spectra of PVA and all GFPGER-modified PVA samples. High resolution of carbon on plain PVA **(B)**, GFPGER_0_ **(C)**, and GFPGER_120_ **(D)** samples.

## References

[B1] Alberio L. Dale G. L. (1998). Flow cytometric analysis of platelet activation by different collagen types present in the vessel wall. *Br. J. Haematol.* 102 1212–1218. 10.1046/j.1365-2141.1998.00923.x 9753047

[B2] Anderson D. E. J. Hinds M. T. (2012). Extracellular matrix production and regulation in micropatterned endothelial cells. *Biochem. Biophys. Res. Commun.* 427 159–164. 10.1016/j.bbrc.2012.09.034 22995321

[B3] Anderson D. E. J. Pohan G. Raman J. Konecny F. Yim E. K. F. Hinds M. T. (2018). Improving surgical methods for studying vascular grafts in animal models. *Tissue Eng. C Methods* 24 457–464. 10.1089/ten.tec.2018.0099 29984616 PMC6088253

[B4] Anderson D. E. J. Truong K. P. Hagen M. W. Yim E. K. F. Hinds M. T. (2019). Biomimetic modification of poly(vinyl alcohol): Encouraging endothelialization and preventing thrombosis with antiplatelet monotherapy. *Acta Biomater.* 86 291–299. 10.1016/J.ACTBIO.2019.01.008 30639349 PMC6594398

[B5] Bates N. M. Puy C. Jurney P. L. McCarty O. J. T. Hinds M. T. (2020). Evaluation of the effect of crosslinking method of poly(vinyl alcohol) hydrogels on thrombogenicity. *Cardiovasc. Eng. Technol.* 11 448–455. 10.1007/s13239-020-00474-y 32607901 PMC7390681

[B6] Cereceres S. Touchet T. Browning M. B. Smith C. Rivera J. Hö M. (2015). Chronic wound dressings based on collagen-mimetic proteins. *Adv. Wound Care* 4 444–456. 10.1089/wound.2014.0614 26244101 PMC4505774

[B7] Chaouat M. Le Visage C. Baille W. E. Escoubet B. Chaubet F. Mateescu M. A. (2008). A novel cross-linked poly(vinyl alcohol) (PVA) for vascular grafts. *Adv. Funct. Mater.* 18 2855–2861. 10.1002/adfm.200701261

[B8] Cosemans J. M. E. M. Iserbyt B. F. Deckmyn H. Heemskerk J. W. M. (2008). Multiple ways to switch platelet integrins on and off. *J. Thromb. Haemost.* 6 1253–1261. 10.1111/j.1538-7836.2008.03041.x 18513212

[B9] Cutiongco M. F. A. Anderson D. E. J. Hinds M. T. Yim E. K. F. (2015a). In vitro and ex vivo hemocompatibility of off-the-shelf modified poly(vinyl alcohol) vascular grafts. *Acta Biomater.* 25 97–108. 10.1016/j.actbio.2015.07.039 26225735 PMC4762273

[B10] Cutiongco M. F. A. Choo R. K. T. Shen N. J. X. Chua B. M. X. Sju E. Choo A. W. L. (2015b). Composite scaffold of poly(vinyl alcohol) and interfacial polyelectrolyte complexation fibers for controlled biomolecule delivery. *Front. Bioeng. Biotechnol.* 3:3. 10.3389/fbioe.2015.00003 25692128 PMC4315105

[B11] Cutiongco M. F. A. Goh S. H. Aid-Launais R. Le Visage C. Low H. Y. Yim E. K. F. (2016a). Planar and tubular patterning of micro and nano-topographies on poly(vinyl alcohol) hydrogel for improved endothelial cell responses. *Biomaterials* 84 184–195. 10.1016/J.BIOMATERIALS.2016.01.036 26828683

[B12] Cutiongco M. F. A. Kukumberg M. Peneyra J. L. Yeo M. S. Yao J. Y. Rufaihah A. J. (2016b). Submillimeter diameter poly(vinyl alcohol) vascular graft patency in rabbit model. *Front. Bioeng. Biotechnol.* 4:44. 10.3389/fbioe.2016.00044 27376059 PMC4896917

[B13] Farndale R. W. (2006). Collagen-induced platelet activation, Blood Cells. *Mol. Dis.* 36 162–165. 10.1016/j.bcmd.2005.12.016 16464621

[B14] Fayol D. Le Visage C. Ino J. Gazeau F. Letourneur D. Wilhelm C. (2013). Design of biomimetic vascular grafts with magnetic endothelial patterning. *Cell Transplant.* 22 2105–2118. 10.3727/096368912X661300 23295155

[B15] Gabriel M. Dahm M. Vahl C. F. (2011). Wet-chemical approach for the cell-adhesive modification of polytetrafluoroethylene. *Biomed. Mater.* 6:035007 10.1088/1748-6041/6/3/035007 21505229

[B16] Glynn J. J. Hinds M. T. (2014). Endothelial outgrowth cells: function and performance in vascular grafts. *Tissue Eng. Part B* 20 294–303. 10.1089/ten.teb.2013.0285 24004404 PMC4123462

[B17] Gupta A. Johnston C. M. Hinds M. T. Anderson D. E. J. (2020). Quantifying physical thrombus characteristics on cardiovascular biomaterials using microCT. *Methods Protoc.* 3:29. 10.3390/mps3020029 32295060 PMC7359709

[B18] Hagen M. W. Hinds M. T. (2019). Static spatial growth restriction micropatterning of endothelial colony forming cells influences their morphology and gene expression. *PLoS One* 14:e0218197. 10.1371/journal.pone.0218197 31188903 PMC6561595

[B19] Harrison S. Vavken P. Kevy S. Jacobson M. Zurakowski D. Murray M. M. (2011). Platelet activation by collagen provides sustained release of anabolic cytokines. *Am. J. Sports Med.* 39 729–734. 10.1177/0363546511401576 21398575 PMC3176726

[B20] Ino J. M. Sju E. Ollivier V. Yim E. K. F. Letourneur D. Le Visage C. (2013). Evaluation of hemocompatibility and endothelialization of hybrid poly(vinyl alcohol) (PVA)/gelatin polymer films. *J. Biomed. Mater. Res. B Appl. Biomater.* 101 1549–1559. 10.1002/jbm.b.32977 23846987

[B21] Jurney P. L. Anderson D. E. J. Pohan G. Yim E. K. F. Hinds M. T. (2018). Reactive ion plasma modification of poly(vinyl-alcohol) increases primary endothelial cell affinity and reduces thrombogenicity. *Macromol. Biosci.* 18:1800132. 10.1002/mabi.201800132 30256533 PMC6644031

[B22] Kumar V. A. Brewster L. P. Caves J. M. Chaikof E. L. (2011). Tissue engineering of blood vessels: functional requirements, progress, and future challenges. *Cardiovasc. Eng. Technol.* 2 137–148. 10.1007/s13239-011-0049-3 23181145 PMC3505086

[B23] Lei Y. Remy M. Labrugere C. Durrieu M. C. (2012). Peptide immobilization on polyethylene terephthalate surfaces to study specific endothelial cell adhesion, spreading and migration. *J. Mater. Sci. Mater. Med.* 23 2761–2772. 10.1007/s10856-012-4736-x 22878726

[B24] Li J. Ding M. Fu Q. Tan H. Xie X. Zhong Y. (2008). A novel strategy to graft RGD peptide on biomaterials surfaces for endothelization of small-diamater vascular grafts and tissue engineering blood vessel. *J. Mater. Sci. Mater. Med.* 19 2595–2603. 10.1007/s10856-007-3354-5 18197370

[B25] Liu T. Liu S. Zhang K. Chen J. Huang N. (2014). Endothelialization of implanted cardiovascular biomaterial surfaces: The development from in vitro to in vivo. *J. Biomed. Mater. Res. A* 102 3754–3772. 10.1002/jbm.a.35025 24243819

[B26] McCarty O. J. T. Larson M. K. Auger J. M. Kalia N. Atkinson B. T. Pearce A. C. (2005). Rac1 is essential for platelet lamellipodia formation and aggregate stability under flow. *J. Biol. Chem.* 280 39474–39484. 10.1074/jbc.M504672200 16195235 PMC1395485

[B27] Munnix I. C. A. Gilio K. Siljander P. R. M. Raynal N. Feijge M. A. H. Hackeng T. M. (2008). Collagen-mimetic peptides mediate flow-dependent thrombus formation by high- or low-affinity binding of integrin α2β1 and glycoprotein VI. *J. Thromb. Haemost.* 6 2132–2142. 10.1111/j.1538-7836.2008.03167.x 18826391

[B28] Munoz-Pinto D. J. Guiza-Arguello V. R. Becerra-Bayona S. M. Erndt-Marino J. Samavedi S. Malmut S. (2015). Collagen-mimetic hydrogels promote human endothelial cell adhesion, migration and phenotypic maturation. *J. Mater. Chem. B Mater. Biol. Med.* 3 7912–7919. 10.1039/C5TB00990A 28989705 PMC5629973

[B29] Nakayama Y. Ishibashi-Ueda H. Takamizawa K. (2004). *In Vivo Tissue-Engineered Small-Caliber Arterial Graft Prosthesis Consisting of Autologous Tissue (Biotube).* Available online at: www.cognizantcommunication.com (accessed April 9, 2020) 10.3727/000000004783983828 15468686

[B30] Nuttelman C. R. Mortisen D. J. Henry S. M. Anseth K. S. (2001). Attachment of fibronectin to poly(vinyl alcohol) hydrogels promotes NIH3T3 cell adhesion, proliferation, and migration. *J. Biomed. Mater. Res.* 57 217–223. 10.1002/1097-4636(200111)57:2<217::aid-jbm1161>3.0.co;2-i 11484184

[B31] Rich R. L. Deivanayagam C. C. S. Owens R. T. Carson M. Höök A. Moore D. (1999). Trench-shaped binding sites promote multiple classes of interactions between collagen and the adherence receptors, α1β1 integrin and Staphylococcus aureus Cna MSCRAMM. *J. Biol. Chem.* 274 24906–24913. 10.1074/jbc.274.35.24906 10455165

[B32] Rizwan M. Yao Y. Gorbet M. B. Tse J. W. Anderson D. E. J. Hinds M. T. (2020). One-pot covalent grafting of gelatin on poly(vinyl alcohol) hydrogel to enhance endothelialization and hemocompatibility for synthetic vascular graft applications. *ACS Appl. Bio Mater.* 3 693–703. 10.1021/acsabm.9b01026 PMC7351135 32656504

[B33] Ruggeri Z. M. Mendolicchio G. L. (2007). Adhesion mechanisms in platelet function. *Circ. Res.* 100 1673–1685. 10.1161/01.RES.0000267878.97021.AB 17585075

[B34] Schindelin J. Arganda-Carreras I. Frise E. Kaynig V. Longair M. Pietzsch T. (2012). Fiji: an open-source platform for biological-image analysis. *Nat. Methods* 9 676–682. 10.1038/nmeth.2019 22743772 PMC3855844

[B35] Schmedlen R. H. Masters K. S. West J. L. (2002). Photocrosslinkable polyvinyl alcohol hydrogels that can be modified with cell adhesion peptides for use in tissue engineering. *Biomaterials* 23 4325–4332. 10.1016/S0142-9612(02)00177-1 12219822

[B36] Seo N. Russell B. H. Rivera J. J. Liang X. Xu X. Afshar-Kharghan V. (2010). An engineered α1 integrin-binding collagenous sequence. *J. Biol. Chem.* 285 31046–31054. 10.1074/jbc.M110.151357 20675378 PMC2945595

[B37] Sipilä K. H. Drushinin K. Rappu P. Jokinen J. Salminen T. A. Salo A. M. (2018). Proline hydroxylation in collagen supports integrin binding by two distinct mechanisms. *J. Biol. Chem.* 293 7645–7658. 10.1074/jbc.RA118.002200 29615493 PMC5961056

[B38] Sivaraman B. Latour R. A. (2010). The relationship between platelet adhesion on surfaces and the structure versus the amount of adsorbed fibrinogen. *Biomaterials* 31 832–839. 10.1016/j.biomaterials.2009.10.008 19850334 PMC2790000

[B39] Uchida N. Emoto H. Kambic H. Harasaki H. Chen J.-F. Hsu S.-H. (1989). Compliance effect on patency of small diameter. *Trans. Am. Soc. Artif. Intern. Organs* 35 556–558. 10.1097/00002480-198907000-00124 2597533

[B40] Vaníčková M. Suttnar J. Dyr J. E. (2006). The adhesion of blood platelets on fibrinogen surface: comparison of two biochemical microplate assays. *Platelets* 17 470–476. 10.1080/09537100600758875 17074723

[B41] Xu Y. Gurusiddappa S. Rich R. L. Owens R. T. Keene D. R. Mayne R. (2000). Multiple binding sites in collagen type I for the integrins α1β1 and α2β1. *J. Biol. Chem.* 275 38981–38989. 10.1074/jbc.M007668200 10986291

[B42] Yao Y. Zaw A. M. Anderson D. E. J. Hinds M. T. Yim E. K. F. (2020). Fucoidan functionalization on poly(vinyl alcohol) hydrogels for improved endothelialization and hemocompatibility. *Biomaterials* 249:120011. 10.1016/j.biomaterials.2020.120011 32304872 PMC7748769

[B43] Zilla P. Bezuidenhout D. Human P. (2007). Prosthetic vascular grafts: wrong models, wrong questions and no healing. *Biomaterials* 28 5009–5027. 10.1016/j.biomaterials.2007.07.017 17688939

