# Supplementary figures and images for: Bioconjugation of a Collagen-Mimicking Peptide Onto Poly(vinyl alcohol) Encourages Endothelialization While Minimizing Thrombosis
Source: Front Bioeng Biotechnol. 2020 Dec 18;8:621768. doi: 10.3389/fbioe.2020.621768 (PMC7793657; doi:10.3389/fbioe.2020.621768)

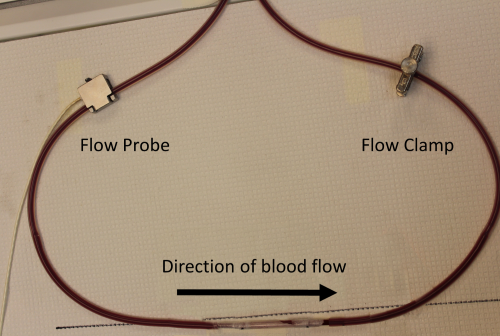

Supplement: Supplementary Figure 1 — Shunt study set up. A shunt was placed between the femoral artery and the femoral vein of a non-human primate (not pictured) using Silastic tubing. The sample to be tested was placed between the tubing using connectors, such that blood flow was continuous through the ex vivo shunt loop. Whole blood was used in this study without the presence of anticoagulant or antiplatelet therapies. Flow rate was controlled at 100 mL/min using the downstream flow clamp and measured using the upstream flow probe. This figure has been modified to remove all informal labeling using Photoshop. [file Image_1.TIF]

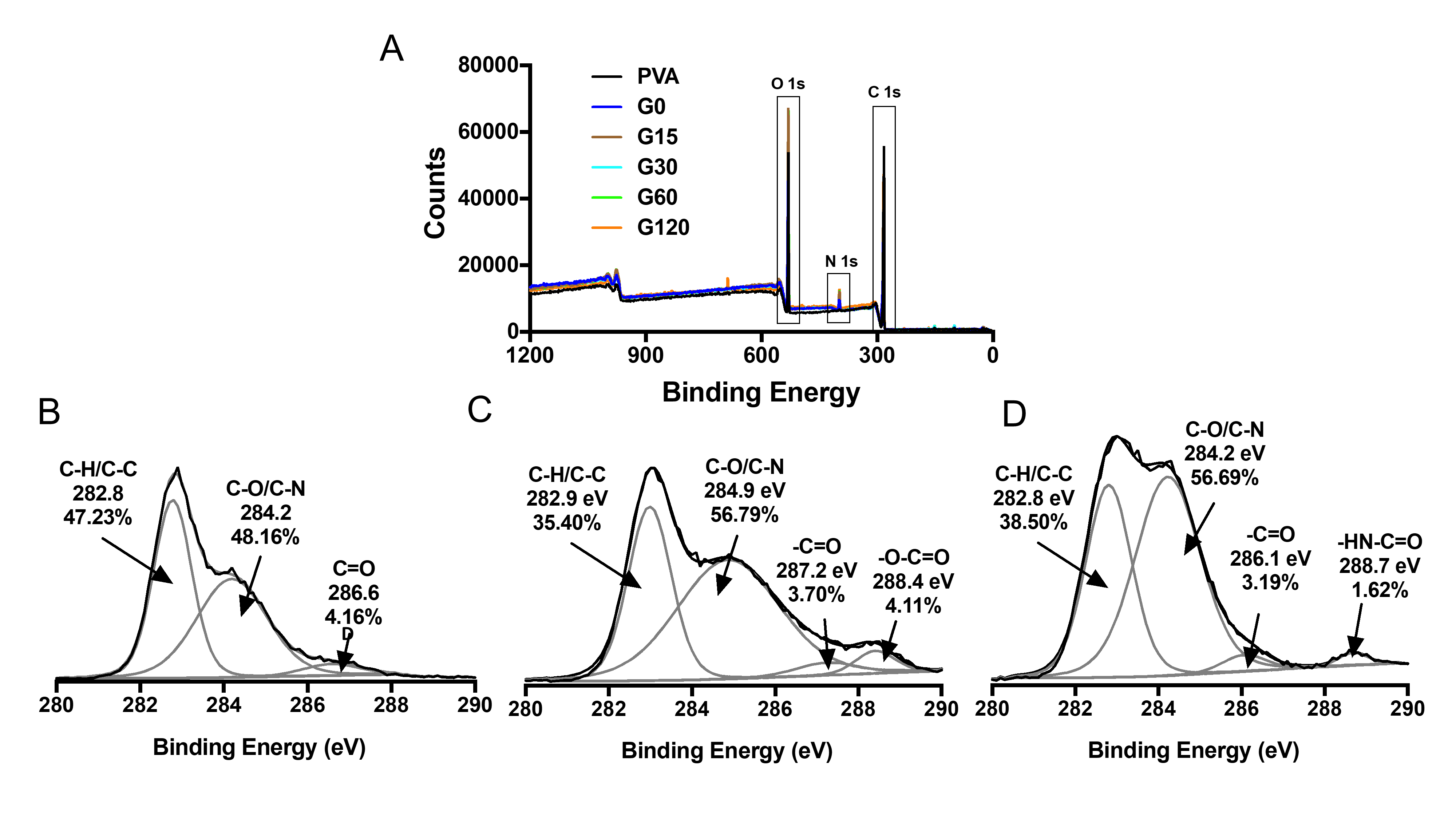

Supplement: Supplementary Figure 2 — (A) Survey XPS spectra of PVA and all GFPGER-modified PVA samples. High resolution of carbon on plain PVA (B), GFPGER0 (C), and GFPGER120 (D) samples. [file Image_2.TIF]
